# Supplementary figures and images for: Structure-guided loop grafting improves expression and stability of influenza neuraminidase for vaccine development
Source: eLife. 2025 Sep 9;14:RP105317. doi: 10.7554/eLife.105317 (PMC12419796; doi:10.7554/eLife.105317)

Reduced Gel 1

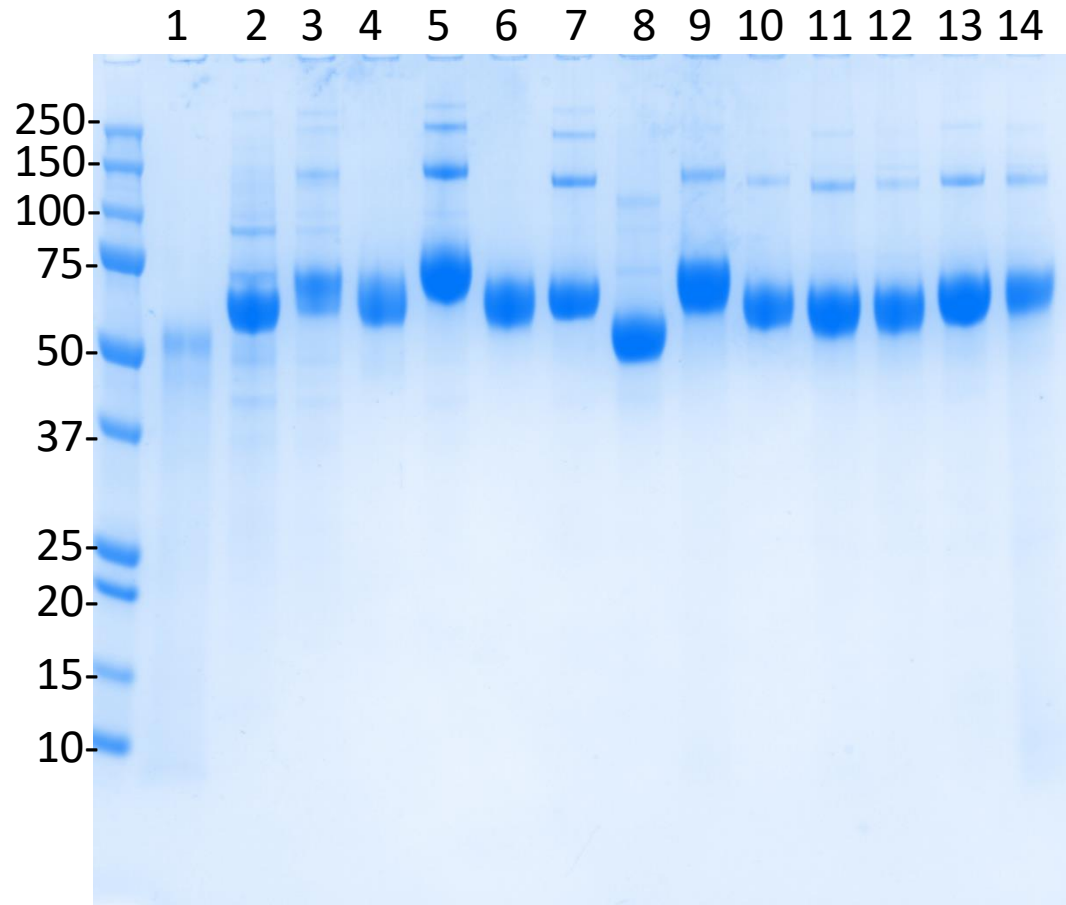

Reduced Gel 2

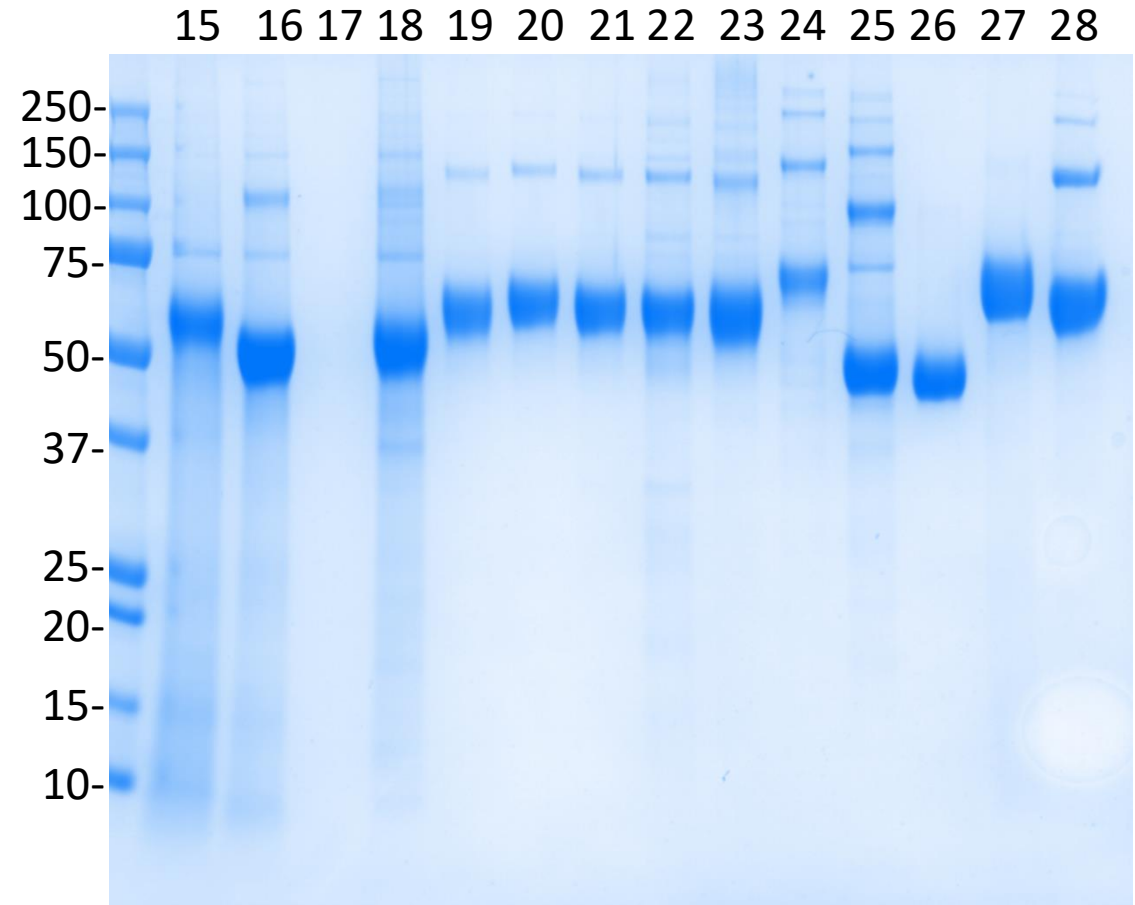

| Lanes | Protein Name |
|-------|--------------|
| 4     | N1/09        |
| 11    | mSN1         |
| 12    | PR8 N1       |
| 19    | N1/09 hybrid |
| 20    | N1/19 hybrid |
| 21    | PR8 hybrid   |
| 22    | mS hybrid    |

Supplement: Figure 3—figure supplement 1—source data 1. [file elife-105317-fig3-figsupp1-data1.zip › Figure 3 - figure supplement 1-Source data 1.pdf]

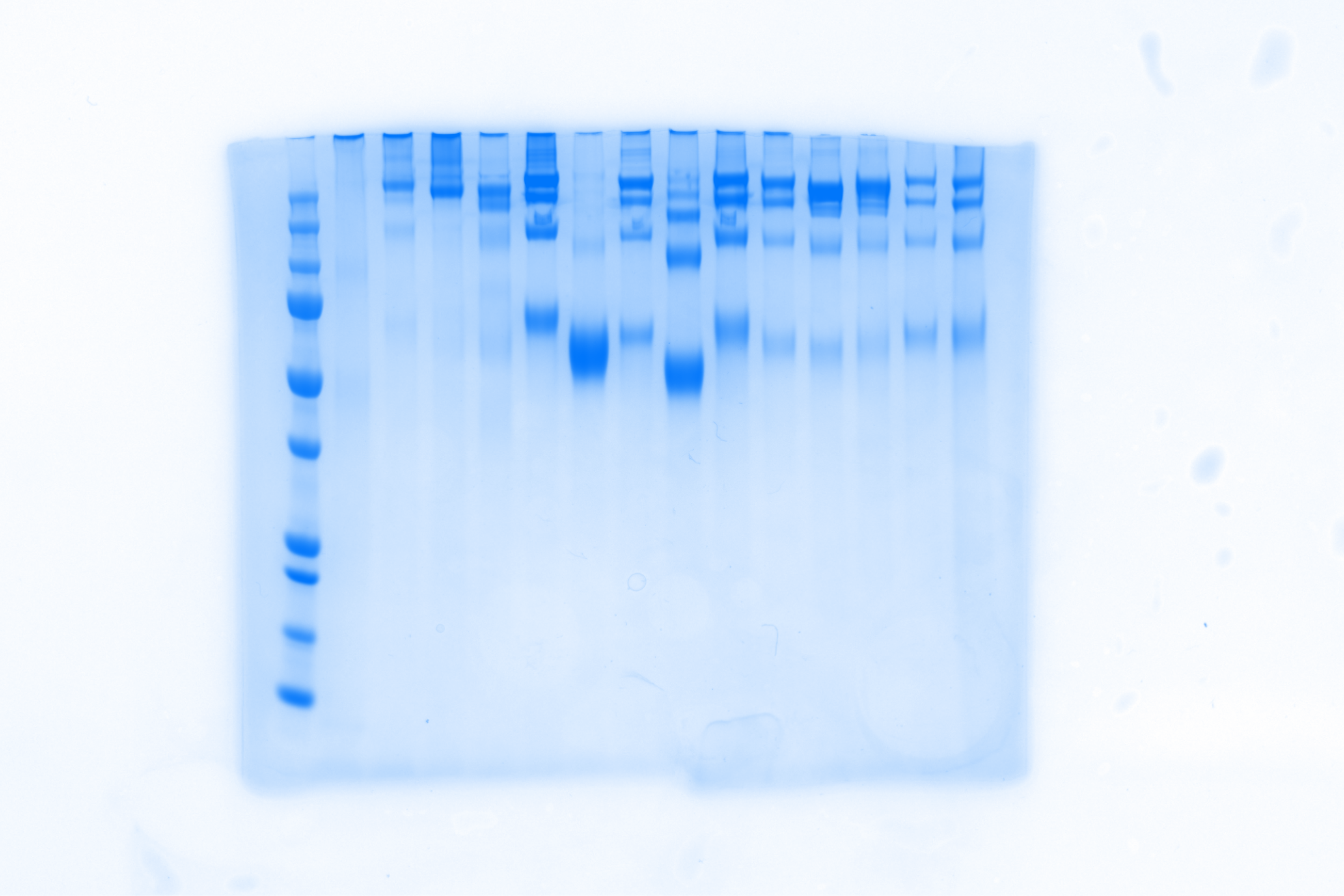

Supplement: Figure 3—figure supplement 1—source data 3. [file elife-105317-fig3-figsupp1-data3.zip › BS3 SDS-PAGE-1.tif]

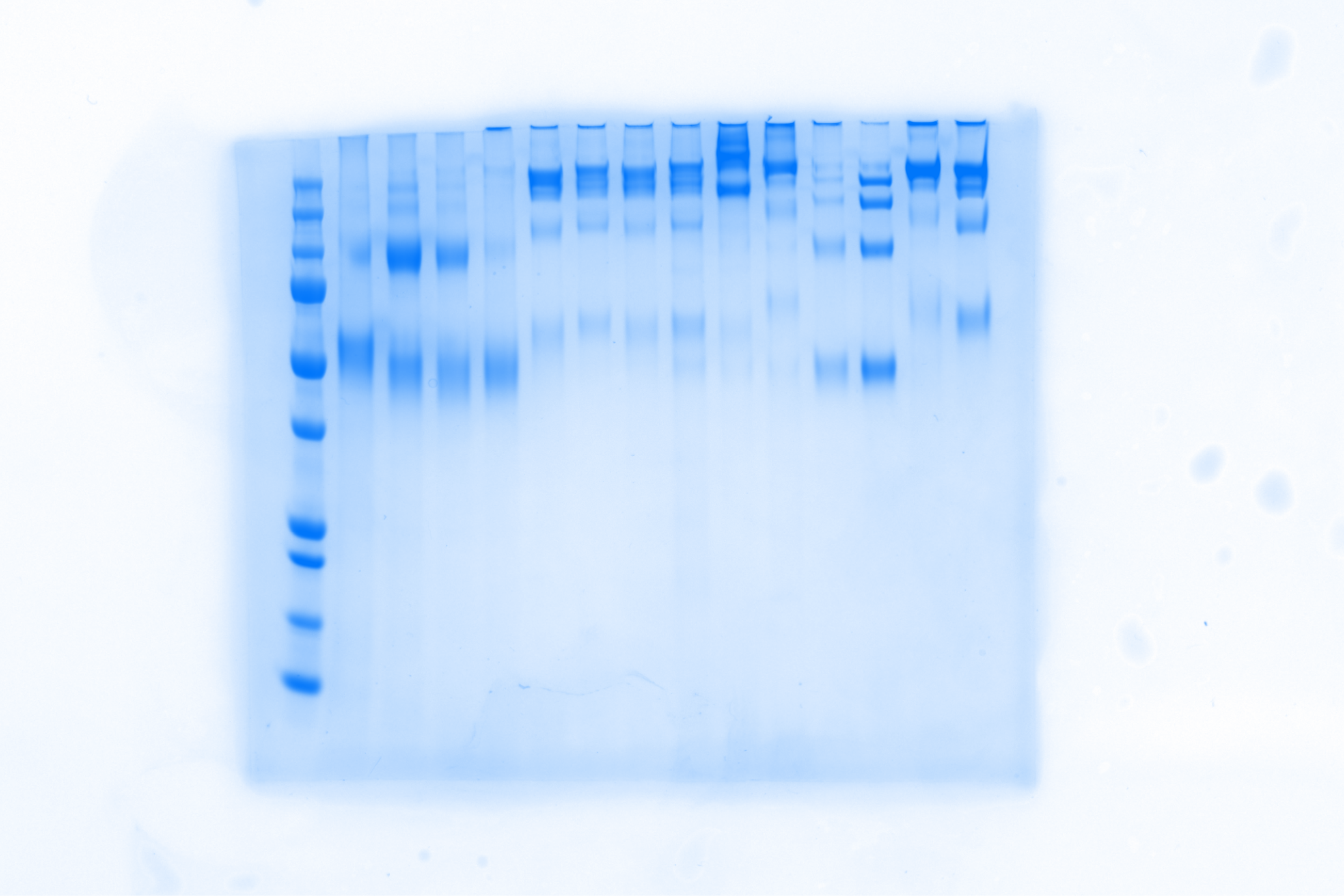

Supplement: Figure 3—figure supplement 1—source data 3. [file elife-105317-fig3-figsupp1-data3.zip › BS3 SDS-PAGE-2.tif]

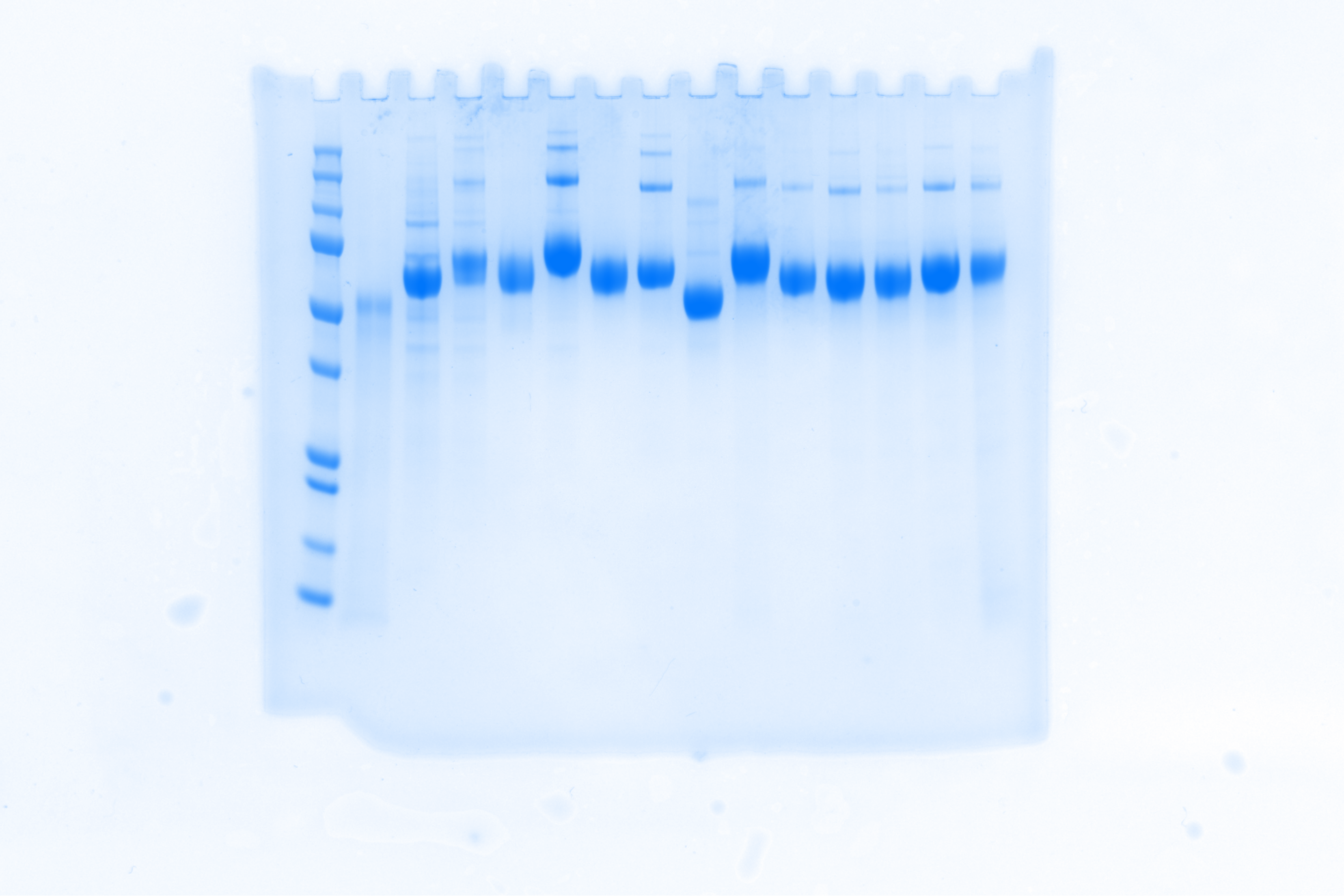

Supplement: Figure 3—figure supplement 1—source data 3. [file elife-105317-fig3-figsupp1-data3.zip › SDS-PAGE 1.tif]

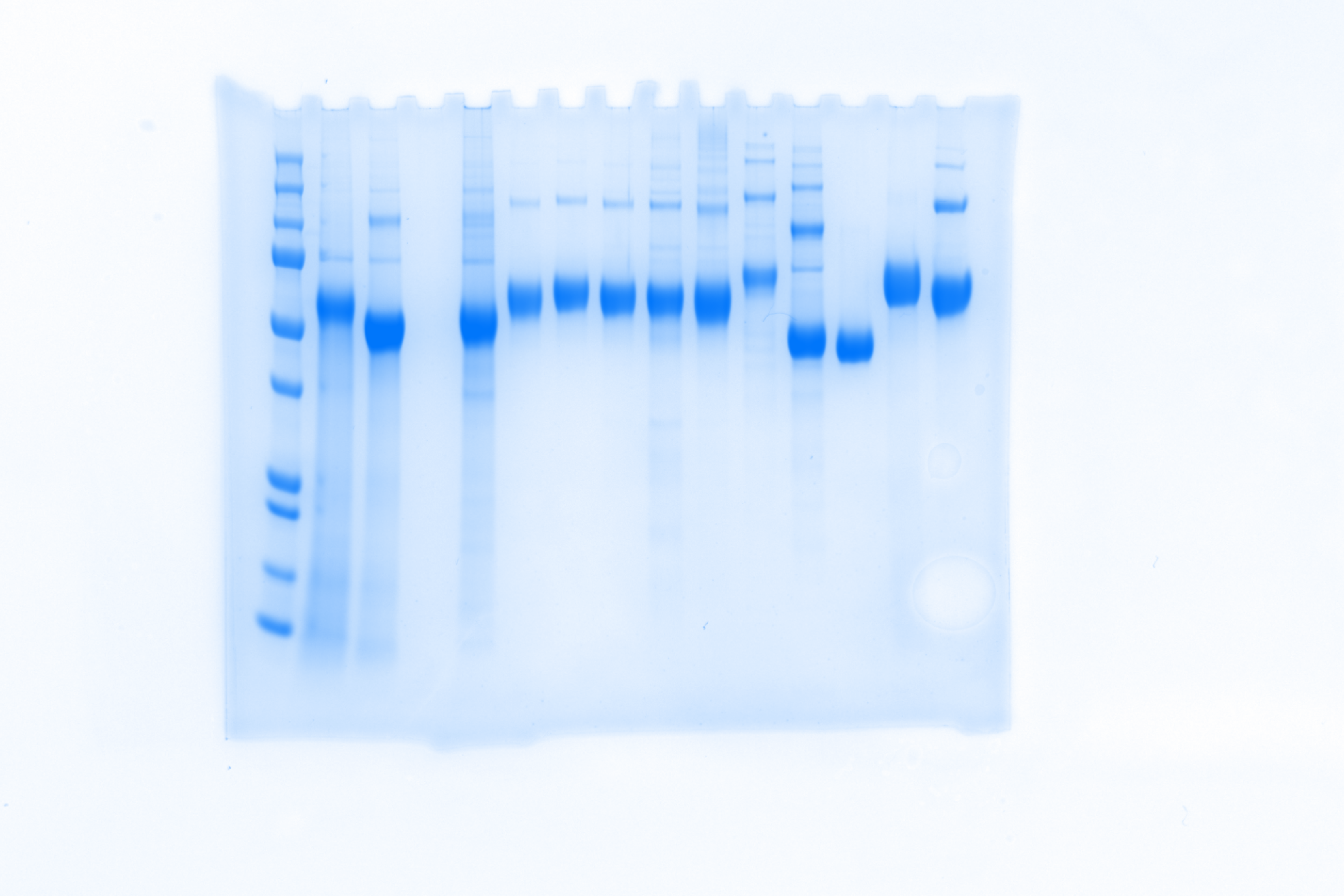

Supplement: Figure 3—figure supplement 1—source data 3. [file elife-105317-fig3-figsupp1-data3.zip › SDS-PAGE 2.tif]
