# Supplementary material for: Structure-guided loop grafting improves expression and stability of influenza neuraminidase for vaccine development
Source: eLife. 2025 Sep 9;14:RP105317. doi: 10.7554/eLife.105317 (PMC12419796; doi:10.7554/eLife.105317)
Supplement: Figure 3—figure supplement 1—source data 2. [file elife-105317-fig3-figsupp1-data2.zip › Figure 3 - figure supplement 1-Source data 2.pdf]

BS-3 cross-linked Gel 1

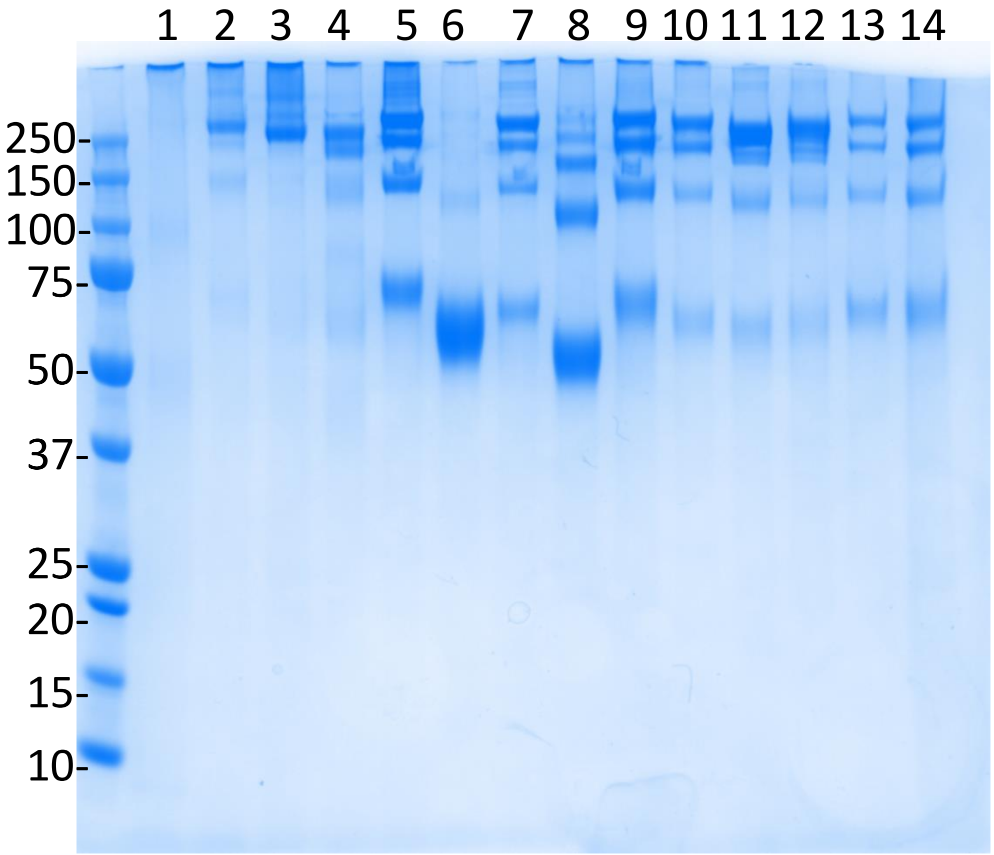

BS-3 cross-linked Gel 2

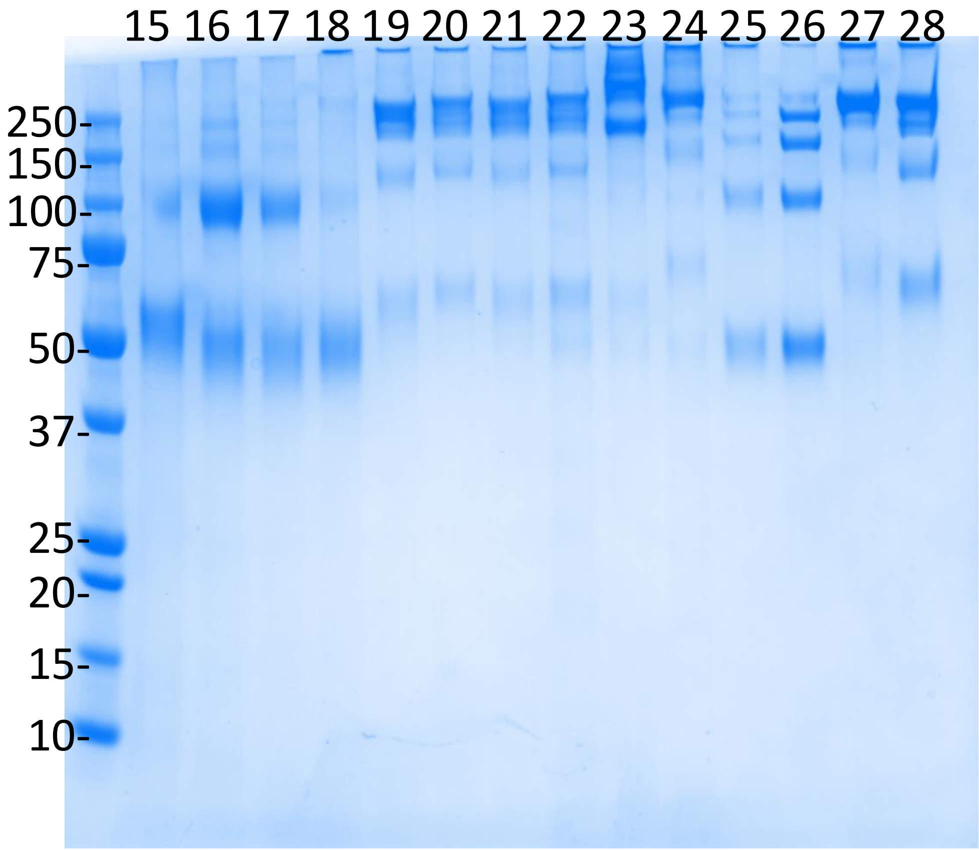

Lanes	Protein Name
4	N1/09
11	mSN1
12	PR8 N1
19	N1/09 hybrid
20	N1/19 hybrid
21	PR8 hybrid
22	mS hybrid
