# Supplementary material for: Structure-guided loop grafting improves expression and stability of influenza neuraminidase for vaccine development
Source: eLife. 2025 Sep 9;14:RP105317. doi: 10.7554/eLife.105317 (PMC12419796; doi:10.7554/eLife.105317)
Supplement: Supplementary file 1. [file elife-105317-supp1.docx]

| Literature | Subtype (NA) | Virus type | Expression System | Yield |
| --- | --- | --- | --- | --- |
| Prevato 2015 ^1^ | N1 | H1N1/09 | Expi293F | **30 mg/L** |
| Ellis 2022 ^2^ | N1, N2, N8 | H1N1/09, H1N1/15, H3N2/05, H10N8/13 | Expi293F | **up to 30 mg/L** |
| Ecker 2020 ^3^ | N1, N2 | H1N1/09 H1N1/18, H3N2/13, H3N2/14, H3N2/16 | Expi293F | **up to 28 mg/L** |
| Prevato 2015 ^1^ | N1 | H5N1 | Expi293F | **6 mg/L** |
| Martinet 1997 ^4^ | N2 | H3N2/1975 | *P Pastoris* | **﻿2.5–3 mg/L** |
| Woude 2020 ^5^ | N2 | H3N2 | 293T | **2.5 mg/L** |
| Subathra 2014 ^6^ | N1 | H1N1 | *P Pastoris* | **2 mg/L** |
| Schmidt 2011 ^7^ | N1 | H1N1 | Sf21 | **0.5-1.8 mg/L** |
| Nivitchanyong 2011 ^8^ | N1 | H5N1 | 293-F | **0.3-0.7 mg/L** |
| Margine 2013 ^9^ | N9 | H7N9/2013 | Sf9 | **0.2-0.7 mg/L** |
| Liu 2015 ^10^ | N1 | H1N1/09, H5N1 | Sf9 | **0.25-0.5 mg/L** |
