# Supplementary material for: Structure-guided loop grafting improves expression and stability of influenza neuraminidase for vaccine development
Source: eLife. 2025 Sep 9;14:RP105317. doi: 10.7554/eLife.105317 (PMC12419796; doi:10.7554/eLife.105317)
Supplement: Supplementary file 2. — Loops in mSN1, N1/09, N1/19, and PR8N1 are listed. Text in orange colour where N1 differs from the original N2 numbering system. Residues in the loops that differ from mSN1 sequence are marked in red (‘aa’ denotes amino acids). [file elife-105317-supp2.docx]

| **Loops** | **N2 numbering** | **N2 NA Loops (Varghese et al. 1983)** | **N1 numbering** | **mSN1 Loops** | **N1/09 Loops** | | **N1/19 Loops** | | **PR8 N1 Loops** | | |  |
| --- | --- | --- | --- | --- | --- | --- | --- | --- | --- | --- | --- | --- |
| **B1L01** | 107-119 | RLSAGGDIWVTRE | 107-119 | RIGSKGDVFVIRE | RIGSKGDVFVIRE | | RIGSKGDVFVIRE | | RIGSKGDVFVIRE | | |  |
| **B1L23** | 135-156 | GQGTTLDNKHSNDTVHDRIPHR | 135-156 | TQGALLNDKHSNGTVKDRSPYR | TQGALLNDKHSNGTIKDRSPYR | | TQGALLNDKHSNGTIKDRSPYR | | TQGALLNDRHSNGTVKDRSPYR | | |  |
| **B2L01** | 175-177 | CIA | 176-178 | SVA | SVA | | SVA | | SVA | | |  |
| **B2L23** | 195-199 | TGDDK | 196-199 | SGPD | SGPD | | SGPD | | SGPD | | |  |
| **B3L01** | 218-227 | WSQNILRTQE | 219-228 | WRNNILRTQE | WRNNILRTQE | | WRNKILRTQE | | WRKKILRTQE | | |  |
| **B3L23** | 243-250 | DGSASGRA | 244-251 | DGPSNGQA | DGPSNGQA | | DGPSDGQA | | DGPSDGLA | | |  |
| **B4L01** | 269-277 | AGSAQHVEE | 269-278 | LNAPNYHYEE | MNAPNYHYEE | | MKAPNYHYEE | | LNAPNSHYEE | | |  |
| **B4L23** | 292-295 | RDNW | 293-296 | RDNW | RDNW | | RDNW | | RDNW | | |  |
| **B5L01** | 315-350 | SYVCSGLVGDTPRNDDRSSNSNCRDPNNERGTQGVK | 314-347 | IGYICSGVFGDNPRPNDGTGSCSPMSSNGAYGVK | IGYICSGIFGDNPRPNDKTGSCGPVSSNGANGVK | | MGYICSGVFGDNPRPNDKTGSCGPVSSNGANGVK | | IGYICSGVFGDNPRPKDGTGSCGPVYVDGANGVK | | |  |
| **B5L23** | 367-371 | SKDLR | 364-369 | STSSRS | SISSRN | | SISSRK | | SHSSRH | | |  |
| **B6L01** | 399-403 | DSDNR | 395-399 | EITDW | GINEW | | GINEW | | AMTDW | | |  |
| **B6L23** | 429-437 | GRKQETRVW | 430-437 | RPKENTIW | RPKENTIW | | RPEENTIW | | GRPKEKTIW | | |  |
| Comments | | | | | | 12 aa changes in 5 Loops | | 16 aa changes in 8 Loops | | 18 aa changes in 8 Loops |  | |
