# Supplementary material for: Structure-guided loop grafting improves expression and stability of influenza neuraminidase for vaccine development
Source: eLife. 2025 Sep 9;14:RP105317. doi: 10.7554/eLife.105317 (PMC12419796; doi:10.7554/eLife.105317)
Supplement: Supplementary file 3. [file elife-105317-supp3.docx]

| **DATA COLLECTION** | **mSN1** | **N1/09** | **N1/19** |
| --- | --- | --- | --- |
|  |  |  |  |
| **Beamline** | DLS I24 | DLS I24 | DLS I24 |
| **Wavelength (Å)** | 0.6199 | 0.6199 | 0.6199 |
| **Space Group** | *P* 4 | *P* 4 | *C* 2 2 2_1_ |
| **Cell Dimensions** |  |  |  |
| a, b, c (Å) | 92.2, 92.2, 147.9 | 92.1, 92.1, 146.8 | 115.5, 234.4, 115.7 |
| 𝛼, β, 𝛾 (°) | 90, 90, 90 | 90, 90, 90 | 90, 90, 90 |
| **Resolution range (Å)** | 147.90-1.98 [2.01-1.98] | 41.18-1.94 [1.97-1.94] | 82.31-1.78 [1.98-1.78] |
| **Rmerge** | 0.766 [2.784] | 0.792 [3.109] | 0.367 [1.705] |
| **I/σ (I)** | 2.8 [0.7] | 4.1 [0.7] | 5.4 [1.6] |
| **CC_1/2_** | 0.976 [0.342] | 0.983 [0.282] | 0.988 [0.564] |
| **Completeness (%)** | 100 [99.8] | 99.9 [98.3] | 94.2 [65.2]* |
| **Multiplicity** | 14.2 [14.1] | 13.9 [10.9] | 13.4 [12.9] |
|  |  |  |  |
|  |  | | |
| **REFINEMENT** |  |  |  |
|  |  |  |  |
| **Resolution (Å)** | 92.17-1.98 | 41.18 -1.94 | 64.69-1.78 |
| **No. reflections** | 83,874 | 89,971 | 58,510 |
| **R_work_/R_free_** | 0.211/0.238 | 0.183/0.213 | 0.233/0.267 |
| **No. atoms** |  |  |  |
| protein | 6,296 | 6,279 | 6,164 |
| ligands | 127 | 102 | 116 |
| solvent | 619 | 695 | 474 |
| **Average B-factors** |  |  |  |
| protein | 29.3 | 24.2 | 18.23 |
| ligand | 65.4 | 66.3 | 60.12 |
| solvent | 35.8 | 33,6 | 25.0 |
| **Ramachandran (%)** |  |  |  |
| favoured | 96.21 | 96.32 | 96.22 |
| allowed | 3.55 | 3.68 | 3.58 |
| outlier | 0.24 | 0 | 0.25 |
| **RMS** |  |  |  |
| bond lengths (Å) | 0.003 | 0.013 | 0.004 |
| bond angles (°) | 0.67 | 1.16 | 0.71 |

The values between brackets are for the highest-resolution shell

*Ellipsoidal completeness as determined by STARANISO
