# Supplementary material for: Structure-guided loop grafting improves expression and stability of influenza neuraminidase for vaccine development
Source: eLife. 2025 Sep 9;14:RP105317. doi: 10.7554/eLife.105317 (PMC12419796; doi:10.7554/eLife.105317)
Supplement: Supplementary file 4. [file elife-105317-supp4.docx]

**Amino acid sequences of NA gene constructs**

H7 HA Signal Sequence: *MNTQILVFALIAIIPTNADKI*

IgKappa Signal Sequence: *METDTLLLWVLLLWVPGSTGD*

Strep Tag II: SAWSHPQFEK

SpyTag: AHIVMVDAYKPTK

6His Purification Tag: HHHHHH

Tetrabrachion tetramerisation domain: IINETADDIVYRLTVIIDDRYESLKNLITLRADRLEMIINDNVSTILA

NA head sequence is coloured blue.

>mSN1 (A/mute swan/England/053054/2021)

*MNTQILVFALIAIIPTNADKI*SAWSHPQFEKGGGGSHHHHHHSSSGSGAHIVMVDAYKPTKGGSGGGGSIINETADDIVYRLTVIIDDRYESLKNLITLRADRLEMIINDNVSTILAGGSGTGSVTLAGNSSLCPISGWAIYSKDNGIRIGSKGDVFVIREPFISCSHLECRTFFLTQGALLNDKHSNGTVKDRSPYRTLMSCPVGEAPSPYNSRFESVAWSASACHDGISWLTIGISGPDNGAVAVVKYNGIITDTIKSWRNNILRTQESECACVNGSCFTVMTDGPSNGQASYKIFKIEKGKVVKSVELNAPNYHYEECSCYPDAGDIMCVCRDNWHGSNRPWVSFNQNLEYQIGYICSGVFGDNPRPNDGTGSCSPMSSNGAYGVKGFSFKYGNGVWIGRTKSTSSRSGFEMIWDPNGWTETDSSFSVKQDIVEITDWSGYSGSFVQHPEMTGLDCMRPCFWVELIRGRPKENTIWTSGSSISFCGVNSDTVGWSWPDGAELPFTIDK*

>N1/09 (A/California/07/2009)

*METDTLLLWVLLLWVPGSTGD*HHHHHHGSGAHIVMVDAYKPTKGGGSIINETADDIVYRLTVIIDDRYESLKNLITLRADRLEMIINDNVSTILAGGSGTGISNTNFAAGQSVVSVKLAGNSSLCPVSGWAIYSKDNSVRIGSKGDVFVIREPFISCSPLECRTFFLTQGALLNDKHSNGTIKDRSPYRTLMSCPIGEVPSPYNSRFESVAWSASACHDGINWLTIGISGPDNGAVAVLKYNGIITDTIKSWRNNILRTQESECACVNGSCFTVMTDGPSNGQASYKIFRIEKGKIVKSVEMNAPNYHYEECSCYPDSSEITCVCRDNWHGSNRPWVSFNQNLEYQIGYICSGIFGDNPRPNDKTGSCGPVSSNGANGVKGFSFKYGNGVWIGRTKSISSRNGFEMIWDPNGWTGTDNNFSIKQDIVGINEWSGYSGSFVQHPELTGLDCIRPCFWVELIRGRPKENTIWTSGSSISFCGVNSDTVGWSWPDGAELPFTIDK*

>N1/19 (A/Wisconsin/588/2019)

*MNTQILVFALIAIIPTNADKI*SAWSHPQFEKGGGGSHHHHHHSSSGSGAHIVMVDAYKPTKGGSGGGGSIINETADDIVYRLTVIIDDRYESLKNLITLRADRLEMIINDNVSTILAGGSGTGSVKLAGNSSLCPVSGWAIYSKDNSVRIGSKGDVFVIREPFISCSPLECRTFFLTQGALLNDKHSNGTIKDRSPYRTLMSCPIGEVPSPYNSRFESVAWSASACHDGTNWLTIGISGPDSGAVAVLKYNGIITDTIKSWRNKILRTQESECACVNGSCFTIMTDGPSDGQASYKIFRIEKGKIIKSVEMKAPNYHYEECSCYPDSSEITCVCRDNWHGSNRPWVSFNQNLEYQMGYICSGVFGDNPRPNDKTGSCGPVSSNGANGVKGFSFKYGNGVWIGRTKSISSRKGFEMIWDPNGWTGTDNKFSKKQDIVGINEWSGYSGSFVQHPELTGLNCIRPCFWVELIRGRPEENTIWTSGSSISFCGVDSDIVGWSWPDGAELPFTIDK*

>N1/09 hybrid (N1/09 Loops - mSN1 Scaffold)

*MNTQILVFALIAIIPTNADKI*SAWSHPQFEKGGGGSHHHHHHSSSGSGAHIVMVDAYKPTKGGSGGGGSIINETADDIVYRLTVIIDDRYESLKNLITLRADRLEMIINDNVSTILAGGSGTGSVTLAGNSSLCPISGWAIYSKDNGIRIGSKGDVFVIREPFISCSHLECRTFFLTQGALLNDKHSNGTIKDRSPYRTLMSCPVGEAPSPYNSRFESVAWSASACHDGISWLTIGISGPDNGAVAVVKYNGIITDTIKSWRNNILRTQESECACVNGSCFTVMTDGPSNGQASYKIFKIEKGKVVKSVEMNAPNYHYEECSCYPDAGDIMCVCRDNWHGSNRPWVSFNQNLEYQIGYICSGIFGDNPRPNDKTGSCGPVSSNGANGVKGFSFKYGNGVWIGRTKSISSRNGFEMIWDPNGWTETDSSFSVKQDIVGINEWSGYSGSFVQHPEMTGLDCMRPCFWVELIRGRPKENTIWTSGSSISFCGVNSDTVGWSWPDGAELPFTIDK*

>N1/19 hybrid (N1/19 Loops - mSN1 Scaffold)

*MNTQILVFALIAIIPTNADKI*SAWSHPQFEKGGGGSHHHHHHSSSGSGAHIVMVDAYKPTKGGSGGGGSIINETADDIVYRLTVIIDDRYESLKNLITLRADRLEMIINDNVSTILAGGSGTGSVTLAGNSSLCPISGWAIYSKDNGIRIGSKGDVFVIREPFISCSHLECRTFFLTQGALLNDKHSNGTIKDRSPYRTLMSCPVGEAPSPYNSRFESVAWSASACHDGISWLTIGISGPDNGAVAVVKYNGIITDTIKSWRNKILRTQESECACVNGSCFTVMTDGPSDGQASYKIFKIEKGKVVKSVEMKAPNYHYEECSCYPDAGDIMCVCRDNWHGSNRPWVSFNQNLEYQMGYICSGVFGDNPRPNDKTGSCGPVSSNGANGVKGFSFKYGNGVWIGRTKSISSRKGFEMIWDPNGWTETDSSFSVKQDIVGINEWSGYSGSFVQHPEMTGLDCMRPCFWVELIRGRPEENTIWTSGSSISFCGVNSDTVGWSWPDGAELPFTIDK*

>PR8 N1 (A/PR/8/1934)

*MNTQILVFALIAIIPTNADKI*SAWSHPQFEKGGGGSHHHHHHSSSGSGAHIVMVDAYKPTKGGSGGGGSIINETADDIVYRLTVIIDDRYESLKNLITLRADRLEMIINDNVSTILAGGSGTGSVILTGNSSLCPIRGWAIYSKDNSIRIGSKGDVFVIREPFISCSHLECRTFFLTQGALLNDRHSNGTVKDRSPYRALMSCPVGEAPSPYNSRFESVAWSASACHDGMGWLTIGISGPDNGAVAVLKYNGIITETIKSWRKKILRTQESECACVNGSCFTIMTDGPSDGLASYKIFKIEKGKVTKSIELNAPNSHYEECSCYPDTGKVMCVCRDNWHGSNRPWVSFDQNLDYQIGYICSGVFGDNPRPKDGTGSCGPVYVDGANGVKGFSYRYGNGVWIGRTKSHSSRHGFEMIWDPNGWTETDSKFSVRQDVVAMTDWSGYSGSFVQHPELTGLDCIRPCFWVELIRGRPKEKTIWTSASSISFCGVNSDTVDWSWPDGAELPFTIDK*

>mS hybrid (mSN1 Loops - PR8 Scaffold)

*MNTQILVFALIAIIPTNADKI*SAWSHPQFEKGGGGSHHHHHHSSSGSGAHIVMVDAYKPTKGGSGGGGSIINETADDIVYRLTVIIDDRYESLKNLITLRADRLEMIINDNVSTILAGGSGTGSVILTGNSSLCPIRGWAIYSKDNSIRIGSKGDVFVIREPFISCSHLECRTFFLTQGALLNDKHSNGTVKDRSPYRALMSCPVGEAPSPYNSRFESVAWSASACHDGMGWLTIGISGPDNGAVAVLKYNGIITETIKSWRNNILRTQESECACVNGSCFTIMTDGPSNGQASYKIFKIEKGKVTKSIELNAPNYHYEECSCYPDTGKVMCVCRDNWHGSNRPWVSFDQNLDYQIGYICSGVFGDNPRPNDGTGSCSPMSSNGAYGVKGFSYRYGNGVWIGRTKSTSSRSGFEMIWDPNGWTETDSKFSVRQDVVEITDWSGYSGSFVQHPELTGLDCIRPCFWVELIRGRPKENTIWTSASSISFCGVNSDTVDWSWPDGAELPFTIDK*

>PR8 hybrid (PR8 N1 Loops - mSN1 Scaffold)

*MNTQILVFALIAIIPTNADKI*SAWSHPQFEKGGGGSHHHHHHSSSGSGAHIVMVDAYKPTKGGSGGGGSIINETADDIVYRLTVIIDDRYESLKNLITLRADRLEMIINDNVSTILAGGSGTGSVTLAGNSSLCPISGWAIYSKDNGIRIGSKGDVFVIREPFISCSHLECRTFFLTQGALLNDRHSNGTVKDRSPYRTLMSCPVGEAPSPYNSRFESVAWSASACHDGISWLTIGISGPDNGAVAVVKYNGIITDTIKSWRKKILRTQESECACVNGSCFTVMTDGPSDGLASYKIFKIEKGKVVKSVELNAPNSHYEECSCYPDAGDIMCVCRDNWHGSNRPWVSFNQNLEYQIGYICSGVFGDNPRPKDGTGSCGPVYVDGANGVKGFSFKYGNGVWIGRTKSHSSRHGFEMIWDPNGWTETDSSFSVKQDIVAMTDWSGYSGSFVQHPEMTGLDCMRPCFWVELIRGRPKEKTIWTSGSSISFCGVNSDTVGWSWPDGAELPFTIDK*
